# Community integration of adults with disabilities post discharge from an in-patient rehabilitation unit in the Western Cape

**DOI:** 10.4102/sajp.v73i1.361

**Published:** 2017-10-20

**Authors:** Dietlind Gretschel, Surona Visagie, Gakeemah Inglis

**Affiliations:** 1 Centre of Rehabilitation Studies, Stellenbosch University, South Africa; 2 Physiotherapy Division, Faculty of Health Sciences, Stellenbosch University, South Africa

## Abstract

**Introduction:**

Community integration is an important outcome of rehabilitation, because the ultimate focus of rehabilitation is to enable people to participate in their life roles.

**Aim:**

To determine community integration scores achieved by adults with disabilities post discharge from an in-patient rehabilitation centre in the Western Cape Province.

**Method:**

Fifty-nine individuals participated in this cross-sectional study. Community integration was determined using the Reintegration to Normal Living Index (RNLI). Descriptive analysis of age, gender, medical diagnosis and RNLI scores was performed. Kruskal–Wallis test and *t*-tests were used to determine whether there exists any relationship between age, gender, medical diagnosis and RNLI scores (*p* < 0.05).

**Results:**

Participants’ mean age was 45 (± 15.9) years. Of the study participants, 54% were women. The most common diagnosis was stroke (41%), followed by spinal cord injury (30%). The mean overall RNLI score was 66.3 (± 25.5). Persons with brain trauma (stroke or head injury) had a mean of 60.9 (±20.3); those with spinal cord injury had a mean of 75.2 (± 25.8) and those with peripheral impairments had a mean of 65.5 (± 30.5). The RNLI domains ‘personal relationships’ 73.45 (± 31.6) and ‘presentation of self’ 72.13 (± 35.4) recorded the highest mean scores. The domain ‘work or meaningful activities’ had the lowest mean score 52.54 (± 35.3). ‘Community mobility’ (59.9; ± 34.6) and ‘recreation’ (57.3; ± 37.2) also had mean scores below 60. No statistically significant relationships were found between age, gender and medical diagnosis and RNLI scores.

**Conclusion:**

The relatively low mean scores indicate that participants achieved poor community reintegration.

## Introduction

The Framework and Strategy for Disability and Rehabilitation Services in South Africa 2015–2020 states that rehabilitation services for South Africans should ‘make the vital, practical link between medical treatment and the translation of a person’s restored capacity into a productive and health-promoting social and economic life’ (DoH 2013:6). Community integration is defined as: ‘acquiring/resuming age-/gender-/culture-appropriate roles/statuses/activities, including independence/interdependence in decision making, and productive behaviours performed as part of multivariate relationships with family, friends, and others in natural community settings’ (Dijkers 1998:5). Thus community reintegration relates to the ability to function and perform life roles within a context and should be a key objective of rehabilitation (Mothabeng et al. 2012; WHO 2011). Various authors have described poor community integration amongst South Africans with disabilities (Heap, Lorenzo & Thomas 2009; Maleka et al. 2012; Mudzi, Stewart & Musenge 2013; Schneider & Nkoli 2011).

Community integration is impacted negatively by diseases or trauma, such as stroke, brain injury or spinal cord injury (SCI). Mayo et al. (2002) compared community integration of Canadians with stroke (*n* = 365) to Canadians without stroke (486). They found that 65% of community dwelling stroke survivors experienced limitations or restrictions in one or more domains as measured by the Reintegration to Normal Living Index (RNLI) compared to 21% of their peers. Findings on the extent to which community integration is hampered differ quite substantially between studies. For instance, Pang et al. (2007) found that 89% of participants with stroke (*n* = 63) experienced limitations in one or more RNLI domains. Carter et al. (2000) on the other hand reported that 45% of participants with stroke (*n* = 182) experienced limitations in one or more RNLI domains.

All three studies mentioned above concurred that the RNLI domains ‘community mobility’, ‘travel out of town’, ‘social activities’, ‘recreation’ and ‘work or meaningful activities’ were most affected after a stroke. Similar results were reported by Kim et al. (2013) who used the RNLI to assess community integration of 243 Canadians who suffered traumatic brain injuries. In addition they found that participants scored low on ‘family role’.

Boschen, Tonack and Gargaro (2003) used the RNLI to assess community integration of 100 community residing Canadian adults with SCI. They reported that participants experienced the biggest challenges in the domains of ‘social activities’ and ‘work or meaningful activities’. Whiteneck, Tate and Charlifue (1999) found that Americans who suffered a traumatic SCI experienced the biggest limitations in the mobility and occupation domains. Samuelkamaleshkumar et al. (2010) and Sekaran et al. (2010) studied community integration of persons with SCI in south India and concurred with the authors from North America.

A number of studies that investigated community integration, or concepts related to community integration, of persons living with disabilities in South Africa were identified but none used the RNLI as a measuring instrument. Furthermore none provided overall community integration scores or an indication of the total number of participants who struggled with one or more of the domains related to community integration. They were, however, in agreement that persons with disabilities found community integration challenging. As with international studies, participants experienced the biggest challenges in the domains of:

- participation in social and leisure activities (Cawood & Visagie 2016; Cunningham & Rhoda 2014; Fredericks & Visagie 2013; Godlwana & Stewart 2013; Maleka et al. 2012; Mudzi et al. 2013; Rhoda 2012; Rhoda et al. 2011; Rouillard et al. 2012; Wasserman, De Villiers & Bryer 2009)
- participation in work or meaningful activities (Cawood & Visagie 2016; Fredericks & Visagie 2013; Godlwana & Stewart 2013; Maleka et al. 2012; Rhoda 2012; Rouillard et al. 2012; Wasserman et al. 2009)
- assuming previous family roles and responsibilities (Cunningham & Rhoda 2014; Maleka et al. 2012; Rouillard et al. 2012; Wasserman et al. 2009)
- community mobility (Cawood & Visagie 2016; Cunningham & Rhoda 2014; Fredericks & Visagie 2013; Godlwana & Stewart 2013; Maleka et al. 2012; Mudzi et al. 2013; Rhoda et al. 2011; Rouillard et al. 2012).

It can be concluded that while studies differ on the extent to which impairments impact community integration they concur that community integration of persons with disabilities is poorer than that of their peers. Authors also agree that the domains of community mobility, social and leisure activities, resuming family roles and meaningful activity or work are the most affected irrespective of diagnosis. None of these studies included people with a variety of different disabilities. Thus the aim of this study was to determine community integration scores achieved by adults with various disabilities post discharge from an in-patient rehabilitation centre in the Western Cape Province.

## Methods

A quantitative, cross-sectional, descriptive design was used to determine community integration scores achieved by participants and to identify domains of community reintegration that posed the greatest challenges. The impact of variables such as age, gender and medical diagnosis on community integration was also explored.

Participants had received in-patient rehabilitation at a 156-bed government-funded, specialised rehabilitation centre in the Western Cape Province. Rehabilitation programmes at the centre are provided by professionals following an interdisciplinary teamwork approach (Joseph et al. 2013). The team approaches disability from a bio-psychosocial perspective and aims to address impairments, activity limitations and participation restrictions as well as environmental barriers.

All the study participants resided in their communities at the time of data collection. As the rehabilitation centre admits patients from all over the Western Cape Province and also from the Northern and Eastern Cape provinces, participants’ home environments and communities might differ substantially. Differences include rural and urban settings, access to services and infrastructure.

The study population consisted of the 188 patients who had completed their in-patient rehabilitation and were discharged from the centre between 01 September 2012 and 30 November 2012. Data were collected in July and August 2013. Thus, participants had been home between 7 and 9 months at the time of data collection. Participants had to be 18 years or older and living in the community. Those living in care facilities or nursing homes were excluded as were individuals who had had more than one period of in-patient rehabilitation at the centre. Those unable to complete a questionnaire in English, Afrikaans or Xhosa, the three languages most commonly spoken in the Western Cape Province, were also excluded, as were individuals residing outside the Cape Town Metro Health District who did not have access to a telephone or were unable to complete a telephonic questionnaire because of speech-language or cognitive impairments. Finally, individuals not competent to give informed consent were excluded. Competence to give informed consent was determined by the first author based on information in client folders. Any indication in the folder that the individual might not be competent to give informed consent was followed up with a phone call to the individual’s family. Proxy participants were not used as the RNLI shows poor reliability between individuals with a disability and significant others (Tooth et al. 2003). Of the 188 individuals in the study population, 76 had to be excluded based on the exclusion criteria described in Table 1. Of the 112 remaining participants, 4 declined participation and 49 could not be located telephonically or by means of a home visit. Thus 59 individuals participated in the study of whom 31 (53%) completed the RNLI telephonically and 28 (47%) in person during a home visit.

**TABLE 1:** Number of persons excluded based on exclusion criteria.

Exclusion criteria	Number of individuals
Younger than 18 years	17
Deceased prior to data collection	7
More than one period of in-patient admission	40
Unable to complete questionnaire in English, Afrikaans or Xhosa	1
Unable to give informed consent	6
Discharged to a care facility	5
**Total**	**76**

*Source*: Authors’ own work

Community reintegration was measured with the RNLI, an 11-item index. Each item is rated on a scale of 1–10, where 1 represents minimal reintegration and 10 represents complete reintegration. The 11 items collate to a total score of 110, but can be converted to 100 for ease of interpretation (Wood-Dauphinee & Williams 1987).

The RNLI is sensitive, has high internal consistency (Cronbach’s α of 0.87), as well as construct and concurrent validity during both personal and telephonic administration (Hitzig et al. 2012). Mothabeng et al. (2012:32) found the RNLI to be ‘a true measure of community integration’ in a South African context and reliable in measuring community reintegration (Cronbach’s α of 0.974) for persons living with SCI in South Africa. They also established content validity, construct validity (item loadings ranged from 0.86 to 0.93), item convergent validity (corrected RNLI item-total correlation coefficients ranged from 0.73 to 0.91) and item discriminant validity (Fisher’s z value was 4.45 which is > 1.96, the criterion for z) (Mothabeng et al. 2012).

The RNLI was translated into Afrikaans and Xhosa. The translation and back translation were done by professional translators.

Data were collected by the first author and three research assistants. The first author completed a demographic and medical data sheet for each participant from the centre’s electronic database and patient folders. The research assistants then contacted participants, obtained provisional consent and made an appointment for an interview. During the interview they obtained informed consent, verified the information obtained from the participants’ records and completed the RNLI.

The research assistants’ training by the first author included an introduction to the study, obtaining informed consent, verification of personal and medical data, and completion of the RNLI. To ensure uniformity and limit interrater bias, research assistants could repeat questions but not rephrase or explain them. Any questions or concerns raised by participants were recorded in writing by the research assistants and referred to the first author for follow up.

Data were captured on an Excel spreadsheet and checked for correctness. The 11 items of the RNLI were grouped into 9 domains and 2 subscales according to the item aggregation concerning reintegration to normal living patterns as described by Wood-Dauphinee and Williams (1987). For example, the overarching domain of mobility included mobility related to home, community and travelling out of town.

Scores were converted to a score out of 100 using the formula: (Average of related items or domains - 1) x 100/9. Item, domain, subscale and overall scores are presented by descriptive statistics. A *t*-test or Kruskal–Wallis test was used to determine if the variables age, gender and medical diagnosis had an impact on community integration. A *p*-value of < 0.05 was considered to be statistically significant.

### Ethical considerations

Ethical approval was obtained from the Health Research Ethics Committee at Stellenbosch University (S12/11/293). Permission to access medical records of study participants was obtained from the Western Cape Department of Health and the Chief Executive Officer of the Rehabilitation Centre.

## Results

The study sample consisted of 59 persons with disabilities. The mean age of participants was 45 (± 15.92) with a range of 19–82 years. Slightly more women (54%) than men (46%) participated in the study.

**FIGURE 1:**
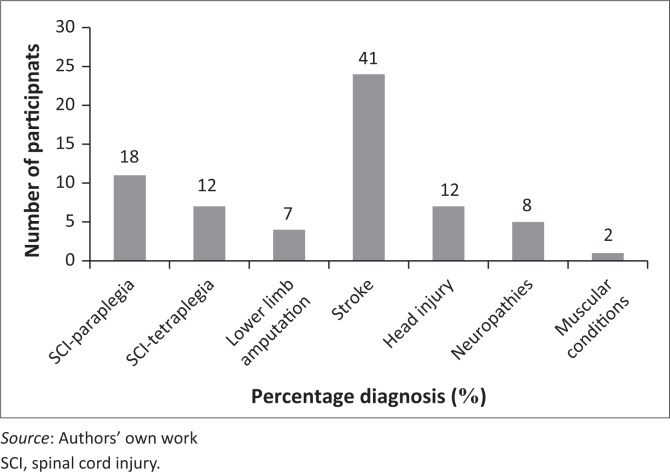
Medical diagnosis of participants (neuropathies included Guillian Barre and retroviral disease-related neuropathies; muscular conditions include muscular dystrophy) (*n* = 59).

Figure 1 shows the various diagnoses, with the most common being stroke (41%) and SCI (30%).

The mean overall RNLI score was 66.3 (± 25.5); ranging from 5.56 to 99.07. The domains ‘personal relationships’ (73.45; ± 31.6) and ‘presentation of self’ (72.13; ± 35.4) recorded the highest mean scores. ‘Work or meaningful activities’ (52.54; ± 35.3) showed the lowest mean. ‘Community mobility’ (59.89; ± 34.6) and ‘recreation’ (57.35; ± 37.2) also had mean scores below 60. The combined mobility scores had a mean of 61.83 (± 27.7). Subscale scores ‘daily functioning’ (61.81; ± 25.2) recorded a lower mean than ‘perception of self’ (70.24; ± 29.6) as illustrated in Table 2.

**TABLE 2:** Descriptive statistics of the Reintegration to Normal Living Index scores (RNLI) (*n* = 59).

RNLI Item	Domain	Mean score	±SD	Minimum	Maximum
Item 1	Indoor mobility	**63.67**	±32.71	0.00	100.00
Item 2	Community mobility	**59.89**	±34.56	0.00	100.00
Item 3	Travel out of town	**62.00**	±35.25	0.00	100.00
**Combined score**	**Mobility**	**61.83**	**±27.71**	**0.00**	**100.00**
Item 4	Self-care	**64.97**	±37.01	0.00	100.00
Item 5	Work/meaningful activities	**52.54**	±35.25	0.00	100.00
Item 6	Recreation	**57.25**	±37.19	0.00	100.00
Item 7	Social activities	**64.97**	±34.39	0.00	100.00
Item 8	Family role	**69.30**	±32.71	0.00	100.00
Item 9	Personal relationships	**73.45**	±31.63	0.00	100.00
Item 10	Presentation of self	**72.13**	±35.40	0.00	100.00
Item 11	Manage life events	**65.16**	±36.53	0.00	100.00
**Subscale**	**Daily functioning**	**61.81**	**±25.24**	**0.00**	**100.00**
**Subscale**	**Perception of self**	**70.24**	**±29.61**	**0.00**	**100.00**
**Overall score**		**66.03**	**±25.51**	**0.00**	**99.07**

*Source*: Authors’ own work

Bold text rows indicate the combined mobility score, subscale scores and the overall score for the RNLI – to distinguish combined scores from RNLI Item scores.

Bold text column indicates highlighting the mean item and combined scores.

The mean scores for men (*n* = 27) and women (*n* = 32) in the various domains were similar with the exception of ‘social activities’ where men scored 8 points lower (*p* = 0.41), and ‘presentation of self’ (*p* = 0.26) where men scored 10 points higher. While the mean scores of ‘daily functioning’ were similar between the two gender groups, that is, 61 (± 26) men versus 62.5 (± 25) women (*p* = 0.83), ‘perception of self’ shows some difference, with women scoring slightly lower in comparison to their male counterparts, that is, 67.7 (± 33) versus 73 (± 25) (*p* = 0.48). None of the differences was statistically significant.

Two age categories were formed, namely 19–49 (*n* = 34) and 50–82 (*n* = 25), to provide larger samples to allow for statistical analysis. The only domain that showed a noticeable difference between the two age categories was ‘work or meaningful activities’. Participants aged between 50 and 82 years scored an average of 58.7 (± 21.4) in comparison to participants in the age category 19–49 years who had an average of 48 (± 28.5) (*p* = 0.26) in this category. The mean scores for the constructs ‘daily functioning’ (60.1; ± 28.4 vs 63.5; ± 20.6) (*p* = 0.67) and ‘perception of self’ (69.7; ± 31.4 vs 71; ± 27.7) (*p* = 0.88), and the overall RNLI (65.2; ± 28.5 vs 67.2; ± 21.4) (*p* = 0.76) score showed little difference between the two age categories.

Similarly, diagnoses were combined to create three broad diagnostic groups, that is, SCI, brain trauma and peripheral impairments:

- SCI (*n* = 18): including paraplegia and tetraplegia. Even though persons with higher level lesions might experience more problems than those with lower level lesions (Boschen et al. 2003; Sekaran et al. 2010) this combination was considered more appropriate than combining persons with SCI with persons who might have cognitive impairments because of brain trauma.
- Brain trauma (*n* = 31): including stroke and head injury. While taking cognisance of the findings by Mayo et al. (2002) and Kim et al. (2013) that individuals with traumatic brain injury experienced greater limitations in most items of the RNLI in comparison to individuals with stroke, the authors decided to combine these diagnostic groups as they are more comparable than to any of the other diagnostic groups because of possible cognitive involvement.
- Peripheral impairments (*n* = 10): including participants with lower limb amputation, neuropathies (including Guillian Barre and retroviral disease related neuropathies) and muscular conditions.

**FIGURE 2:**
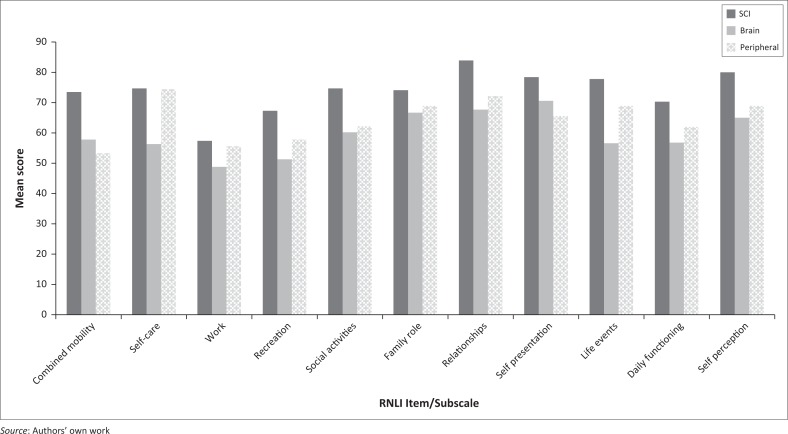
Mean Reintegration to Normal Living Index domain scores for the three diagnostic groups.

The findings indicated that participants with SCI had higher community integration scores in all the individual domains, subscales and total scores of the RNLI. While the SCI group achieved an overall mean score of 75.15 (± 20.3), participants of the brain trauma and peripheral impairments groups had overall mean scores of 60.91 (± 25.8) and 65.46 (± 30.5), respectively (*p* = 0.15). Figure 2 shows that the mean combined ‘mobility’ score for those with SCI (73.5; ± 25.6) was 15.7 points higher than that of the brain trauma group (57.8; ± 27.2) and 20.2 points higher than that of the peripheral impairment group (53.3; ± 29.0). Participants with peripheral impairments scored the lowest in the ‘combined mobility’.

‘Work or meaningful activities’ showed low mean scores for all three groups: brain trauma 48.75 (± 34.2), SCI 57.41 (± 35.6) and peripheral impairments 55.56 (± 40.2). In contrast, ‘relationships’ showed high mean scores for all three groups: brain trauma 67.74 (± 34.5), SCI 84 (± 20.6) and peripheral impairments 72.2 (± 36.8). Participants with brain trauma had a much lower mean score (56.3; ± 37.7) for ‘self-care’ than the other two groups: SCI 74.7 (± 35.7) and peripheral impairments 74.4 (± 33.6).

## Discussion

The RNLI was chosen as the data collection instrument as it is a generic tool that allows collection and thus comparison of information across various health conditions. The overall and diagnostic category mean scores showed that participants achieved poor community integration. This was especially true for ‘overall mobility’, ‘work or meaningful activities’, ‘recreation’ and ‘social activities’. Those with SCI fared somewhat better than participants with peripheral impairments and brain trauma.

The overall mean score of 66.03 and the mean score of the brain trauma group of 60.91 is almost 20 points lower than the 83.3 and 83.1 reported by Carter et al. (2000) and Pang et al. (2007), respectively, in studies conducted with stroke survivors in the United States. Apart from the differences in setting and medical diagnosis, differences in methodologies must be taken into consideration. Carter et al. (2000) and Pang et al. (2007) assessed study participants, on average, 2.75 and 5.5 years after the onset of the stroke, respectively, while in our study participants had only been living with their condition for about a year. The longer period of living with a disability might have assisted participants to adapt to their impairments and find ways to enhance community integration.

Pang et al. (2007) used a four-point ordinal scale and converted scores to a score out of 100. They also excluded wheelchair users. Being dependent on a wheelchair for mobility creates community access challenges that can influence not only the ‘mobility’ domain, but also many others such as ‘recreation’, ‘social activities’ and ‘work or meaningful activities’, all domains in which the participants in our study had lower scores. In the study by Carter et al. (2000), 77% of participants were stroke survivors who reported no residual physical limitations. Physical limitations can negatively impact all aspects of the ‘daily functioning’ subscale, which had a relatively low mean score of just over 60 here.

Obembe et al. (2013) who studied 90 community dwelling Nigerian stroke survivors reported a mean score of 57.3 (±23.5) (range: 39–90). Unfortunately, Obembe et al. (2013) did not provide methodological information that can be used to explore possible reasons for the difference. A possible explanation for the lower scores in the African studies might be the severity and type of environmental barriers experienced. One can expect these barriers to be more prevalent in African countries such as South Africa and Nigeria than in the United States.

The RNLI domains that our study participants perceived to be most challenging, that is, ‘work or meaningful activities, ‘recreation’, ‘social activities’, ‘indoor mobility’, ‘community mobility’ and ‘travel out of town’ were similar to those found in international studies with stroke survivors (Carter et al. 2000; Kim et al. 2013; Mayo et al. 2002) and people with SCI (Samuelkamaleshkumar et al. 2010; Sekaran et al. 2010; Whiteneck et al. 1999). The findings are also in agreement with findings from other South African studies (Cunningham & Rhoda 2014; Fredericks & Visagie 2013; Godlwana & Stewart 2013; Hassan, Visagie & Mji 2011; Maleka et al. 2012; Mudzi et al. 2013; Rouillard et al. 2012; Wasserman et al. 2009). These domains all impact the ‘daily functioning’ subscale and might explain why this subscale showed a lower mean than the ‘perception of self’ subscale.

That finding that the ‘perception of self’ subscale had a mean of almost 10% higher than the ‘daily function subscale’ was surprising. Higher scores in this category are also in contrast with findings from other studies (Cunningham & Rhoda 2014; Kim et al. 2013; Maleka et al. 2012; Mudzi et al. 2013; Rouillard et al. 2012). A higher prevalence of cognitive impairments could have been experienced by study participants in these studies, as unlike in our study participants all had a diagnosis which might involve cognitive impairment. This might have contributed to lower levels of satisfaction with ‘personal relationships’ and ‘perception of self’. The exclusion of at least some participants with cognitive dysfunction might also have lessened the likelihood of a similar result. It might also be that interventions and counselling provided by members of the interdisciplinary team, which include a social worker, during in-patient rehabilitation, assisted participants to develop coping skills and a positive self-image.

The finding that women and men had very similar levels of community integration is similar to findings by Pang et al. (2007) as well as Obembe et al. (2013) and Hamzat et al. (2014) who both studied stroke survivors in Nigeria, but in contradiction to findings by Chau et al. (2009). Chau et al. reported that women who had suffered a stroke experienced lower levels of community participation than men, that they achieved lower self-esteem scores and were also less likely to participate in social and recreational activities. Chau et al. argue that this finding might be influenced by the large value women placed on appearance and body image. Our findings that men scored higher in the category ‘being comfortable with self in the company of others’ than women might provide some support to their argument.

The overall RNLI scores between the two age categories (19–49 and 50–58 years) showed little difference (2.05%). This finding is in contrast with findings from Whiteneck et al. (1999) and Boschen et al. (2003) who reported that younger individuals experienced higher levels of community integration. ‘Work or meaningful activities’ showed the biggest difference between the two age groups. The younger group scored 10.63% lower than the older group in this area. Unemployment might have a bigger impact on participants aged 19 and 49 years who are still in their economically active years. This group is usually employed and building a career, compared to the group older than 50 years who might be preparing for retirement.

‘Work or meaningful activities’ also had the lowest mean score across all diagnostic groups, with participants in the brain trauma group scoring the lowest in this domain. We know from previous studies that persons with disabilities struggle to find employment in South Africa (Cramm, Lorenzo & Nieboer 2013). The reasons for this are complex and involve general high unemployment rates in South Africa, and a myriad of environmental barriers such as poor physical access to environments and transportation, negative attitudes of others and negative assumptions regarding the costs associated with employing persons with disabilities, poor reasonable accommodation, lack of skills and low levels of education, as well as limited access to information regarding employment opportunities and skills training because of high levels of social isolation experienced by persons with disabilities (Schneider & Nkoli 2011).

Interestingly persons with SCI scored higher in all of the domains than participants from the other two diagnostic groups. Information on aspects such as wheelchair dependence for mobility, and length of stay, which were not collected here, is necessary to better understand this finding. With regard to mobility it may be that after SCI a wheelchair is routinely prescribed for any person who cannot walk or walks with difficulty. This might not be the case with brain trauma and some peripheral impairments such as Guillian Barre and neuropathies where some improvement in mobility might still be expected.

The influence of length of stay during in-patient rehabilitation on community integration was not determined, but it is possible that increased length of stay may result in higher levels of community integration (Hastings, Ntsiea & Olorunju 2015; Pezzin et al. 2000) as experienced by participants with SCI. Statistics drawn from the electronic database of the centre show that the average length of stay for in-patients varies from 28 (patients with brain trauma) to 90 days or longer (patients with a high-level SCI). Thus individuals with SCI generally spent an average of 62 days longer in in-patient rehabilitation than individuals who suffered brain trauma.

### Limitations

The relatively low number of study participants (59) might have impacted negatively on comparative analysis and forced pairing of different types of diagnosis, which were less than optimal. Excluding individuals who were unable to give informed consent and individuals residing outside the Cape Town Metro Health District who could not be reached telephonically or were unable to complete the questionnaire telephonically because of cognitive or communication difficulties is a further limitation as these groups might differ from the participants who were studied. Cognitive and communication impairments bring their own challenges when attempting to integrate in the community. Not determining employment figures of participants was a limitation especially in light of the important role work or meaningful activities play in community integration.

## Conclusion

This study provided an overall community integration score as well as scores for the various domains of the RNLI that can be used for comparison in future. When considering that our participants received in-patient rehabilitation at a specialised rehabilitation unit we conclude that the overall community integration achieved by participants as determined through RNLI scores is low. ‘Personal relationships’, ‘presentation of self’ and ‘family roles’ were the domains in which study participants achieved the highest levels of integration. The greatest challenges were experienced with ‘recreation’, ‘social activities’, overall ‘mobility’ and ‘work or meaningful activities’.

### Recommendations

It is recommended that the rehabilitation teams at the study centre ensure that adequate attention is given to participation in recreational, social and work activities as well as community mobility and travel during rehabilitation.
